# Editorial: Outcome of sepsis and prediction of mortality risk

**DOI:** 10.3389/fmed.2023.1338938

**Published:** 2023-12-19

**Authors:** Elena Munari, Marcos Ferreira Minicucci, Zhong Ming, Massimo Girardis, Stefano Busani

**Affiliations:** ^1^Anesthesia and Intensive Care Unit, University Hospital of Modena Policlinico, University of Modena and Reggio Emilia, Modena, Italy; ^2^Internal Medicine Department, Faculdade de Medicina, Universidade Estadual Paulista (UNESP), Botucatu, Brazil; ^3^Department of Critical Care Medicine, Zhongshan Hospital Fudan University, Shanghai, China

**Keywords:** sepsis, outcome, prediction of mortality, ICU, septic shock

Sepsis mortality is a serious concern in healthcare, as it remains one of the leading causes of death worldwide (1). Early identification and prediction of sepsis risk are crucial to improve patient outcomes (2). Advances in machine learning and data analytics have enabled healthcare professionals to develop more accurate predictive models, leveraging patient data to identify those at higher risk (3, 4). Timely intervention and appropriate care can make a significant difference in reducing sepsis mortality rates, highlighting the importance of predictive tools in the battle against this life-threatening condition.

The aim of the Research Topic of the articles in this issue, dedicated to patients with sepsis and septic shock, was to outline some interesting issues on mortality and its risk assessment. Thirteen articles were submitted to this thematic collection, all the articles were original research studies.

It is widely accepted that serum lactate is a parameter of tissue perfusion and represents a marker of sepsis diagnosis. López et al. focused on the lactate trend and made a comparison between septic oncological and non-oncological patients in a retrospective analysis of a prospective database. They showed that hyperlactatemia was associated with higher mortality, and this condition was more frequent in cancer patients than in non-oncological ones (65 vs. 49.1%, *p* = 0.013). In conclusion, immunosuppression due to the malignant disease or its treatment increased the risk for severe infections; lactate levels and poor performance status represented tools for the stratification risk of septic oncological patients. In addition, cancer patients are more exposed to acute kidney injury (AKI) during sepsis, as Yang et al. demonstrated in their retrospective study. Elevated serum lactate levels, high SOFA score and septic shock were strictly related to septic AKI in cancer patients. The 28-day–outcome after ICU admission was worse in oncologic patients with septic AKI than in those without it. Continuous renal replacement therapy, which is an effective treatment for AKI, did not influence the short-term prognosis of cancer patients with septic AKI in the ICU. These considerations could be useful to guide the definition of prognosis and treatment for these critically ill patients. Again, Chen et al. considered hyperlactatemia combined with hypoalbuminemia and patients' age in terms of Lactate/Albumin Ratio and Lactate/Albumin Ratio × Age Score in the assessment of prognosis in patients with sepsis. The statistical analysis showed that the Lac/Alb ratio was an independent risk death factor in septic patients. However, the Lac/Alb × age score was more accurate in the assessment of prognosis, so it could represent a useful tool for clinicians.

In the context of sepsis biomarkers, Peng et al. focused on hyperbilirubinemia and hepatic dysfunction. The propensity score matching showed that septic patients without previous hepatic disease and with total bilirubin (TBIL) levels during ICU admission equal to or more than 5 mg/dl had a higher risk of 1-year mortality than those with TBIL < 5 mg/dl. Moreover, recent studies showed that heparin-binding protein (HBP), a protein in the polymorphonuclear leukocyte, could assess the risk of progression to sepsis with good accuracy. Han et al. showed that serum HBP levels predicted sepsis-related acute organ dysfunction and might improve the accuracy of the qSOFA score. They also have created an online mortality risk calculator that incorporated HBP with qSOFA representing a useful and simple tool to calculate the predicted 30-day mortality.

Concerning inflammatory biomarkers, Li et al. studied the neutrophil/lymphocyte ratio (NLR), a representative parameter of the number of immune cells, associated with in-hospital mortality, and Monocyte/high-density lipoprotein cholesterol ratio (MHR), an indicator of systemic inflammation and oxidative stress. The retrospective analysis of 274 patients showed that high levels of procalcitonin, NLR, and MHR potentially aggravated the 28-day mortality risk of septic patients (*p* < 0.001). In the predictive model of MHR combined with NLR, the AUC maximum value was 0.934 with a better sensitivity and specificity than the single variable. This suggested that these parameters together represented independent risk factors for increased mortality and had predictive efficacies for 28-day mortality risk in septic patients. Again, in this context, many studies showed that decreased lymphocyte count and elevated glucose levels are strongly related to immune dysfunction and the severity of sepsis. These two variables were combined and analyzed in the glucose-to-lymphocyte ratio (GLR) of Cai et al. study on 10,118 patients with sepsis from the MIMIC IV database. Results showed that an elevated GLR was positively related to higher in-hospital mortality in ICU patients with sepsis in the United States, anyway this relationship was not linear. For this reason, further studies are necessary to establish if GLR could have a predictive role in sepsis mortality.

Pieroni et al. studied in-hospital mortality related to the origin of infection. Data were extracted from the eICU collaborative research database covering multi-center ICUs with over 200,000 admissions. The authors considered the three most frequent sources of sepsis: pulmonary, urinary, and abdominal, intending to develop prognostic models for hospital mortality. They made comparisons with the used prediction outcome scores such as APACHE IV and SOFA. They demonstrated that mortality varied significantly between the three sepsis groups with high heterogeneity of the factors that influenced in-hospital mortality. For this reason, the planning of sepsis treatment trials might consider a risk stratification based on the source of infection.

Another reported topic in this research collection was traumatic brain injury (TBI) and septic complications. Caceres et al. focused on lower respiratory tract infections (LRTIs) including hospital-acquired pneumonia, ventilator-associated pneumonia, and ventilator-associated tracheobronchitis. Multivariable analysis showed that age, severe TBI, thorax injuries, and mechanical ventilation on admission to ICU were correlated to the development of LRTIs. Moreover, patients with TBI and a diagnosis of LRTIs had a longer ICU stay and hospital stay and spent more days on mechanical ventilation with no influence on hospital mortality.

Another category of patients reported were severe burn patients, for whom sepsis is one of the main causes of death. Cao et al. made a bibliometric analysis, using the VOSviewer software, that collected the research about burn sepsis using the Web of Science platform, with the aim to establish the global research trends and hotspots in this field. They demonstrated that the treatments of burn sepsis were very different between hospitals worldwide and not standardized. In recent studies, the focus was on biomarkers for early diagnosis of burn sepsis. The hotspots for future research should be the identification of predictive tools for early diagnosis, prognosis, and treatment of burn sepsis using reliable indicators (burn area, biomarkers, etc.).

The early fresh frozen plasma (FFP) transfusion in patients with sepsis or septic shock admitted to ICU were reported by Qin et al.. Medical Information Mart for Intensive Care III database was used for a sensitivity analysis conducted to validate the effects of early FFP transfusion in the patients with sepsis with hypocoagulable and non-hypocoagulable state. They showed that septic patients with hypocoagulable state did not improve their outcomes after early FFP transfusion. Moreover, patients with no hypocoagulable state that received early FFP transfusion increased their mortality risk at 28 and 90 days. For these reasons, it was important to reduce the inappropriate use of FFP to avoid complications and adverse transfusion reactions.

Wedekind et al. developed risk-adjusted quality indicators for the long-term outcome of acute sepsis care in German hospitals based on health claims data on 32,552 patients. A total of 90-day mortality after hospital discharge was chosen as a short-term outcome. As a long-term outcome, they chose a binary outcome of 1-year mortality and an increase in dependency on chronic care during the year after hospital discharge. This health claims-based risk-adjustment methodology could provide a valuable tool in assessing and monitoring outcome quality achieved by German hospitals caring for patients with sepsis, using indicators of long-term mortality and morbidity.

As the final research edited in this collection, Kreitmann et al. analyzed an immune profiling panel prototype, a multiplexed transcriptomic assay that used the array technology to quantify mRNA expression in whole blood and delivered results in less than an hour. In the future, this prototype test could be able to provide clinicians with timely information about the immune system of septic patients and potentially aid in providing care.

Despite the good contribution provided by the 13 articles included in this collection relating to the sepsis outcome, the accurate prediction and assessment of mortality risk associated with sepsis is an area requiring further extensive research due to the absence of standardized tools currently available. The development of reliable methods for predicting and assessing the risk of mortality in sepsis patients remains a crucial and underexplored area in healthcare. As of now, there is a notable absence of universally accepted or standardized tools that effectively gauge the likelihood of mortality in individuals affected by sepsis. Therefore, the urgent need for comprehensive studies arises to establish robust frameworks or methodologies capable of accurately predicting and evaluating the risk of mortality in patients suffering from sepsis.

## Author contributions

EM: Writing—original draft. MM: Writing—review & editing. ZM: Writing—review & editing. MG: Writing—review & editing. SB: Writing—original draft, Writing—review & editing.
